# A link between the fibroblast growth factor axis and the miR‐16 family reveals potential new treatment combinations in mesothelioma

**DOI:** 10.1002/1878-0261.12150

**Published:** 2017-11-18

**Authors:** Karin Schelch, Michaela B. Kirschner, Marissa Williams, Yuen Y. Cheng, Nico van Zandwijk, Michael Grusch, Glen Reid

**Affiliations:** ^1^ Asbestos Diseases Research Institute Sydney Australia; ^2^ Department of Medicine I Institute of Cancer Research Medical University of Vienna Austria; ^3^ Division of Thoracic Surgery University Hospital Zurich Switzerland; ^4^ School of Medicine University of Sydney Australia

**Keywords:** fibroblast growth factor, fibroblast growth factor receptor, malignant pleural mesothelioma, microRNA‐15, microRNA‐16

## Abstract

Malignant pleural mesothelioma (MPM) is an aggressive malignancy with very limited therapeutic options. Fibroblast growth factor (FGF) signals play important roles in mesothelioma cell growth. Several FGFs and FGF receptors (FGFRs) are predicted targets of the miR‐15/16 family, which is downregulated in MPM. The aim of this study was to explore the link between the miR‐15/16 family and the FGF axis in MPM. Expression analyses via RT‐qPCR showed downregulation of the FGF axis after transfection with miR‐15/16 mimics. Direct interaction was confirmed by luciferase reporter assays. Restoration of miR‐15/16 led to dose‐dependent growth inhibition in MPM cell lines, which significantly correlated with their sensitivity to FGFR inhibition. Treatment with recombinant FGF2 prevented growth inhibition and further reduced the levels of FGF/R‐targeting microRNAs, indicating a vicious cycle between miR‐15/16 down‐ and FGF/FGFR signaling upregulation. Combined inhibition of two independent miR‐15/16 targets, the FGF axis and Bcl‐2, resulted in additive or synergistic activity. Our data indicate that post‐transcriptional repression of FGF‐mediated signals contributes to the tumor suppressor function of the microRNA‐15/16 family. Inhibiting hyperactivated FGF signals and Bcl‐2 might serve as a novel therapeutic combination strategy in MPM.

**Abbreviations**
FGF: fibroblast growth factor
FGFR: fibroblast growth factor receptor
MPM: malignant pleural mesothelioma

## 1 Introduction

Malignant pleural mesothelioma (MPM) is a very aggressive malignancy of the pleural linings with dismal outcome (median survival of 9–17 months) and limited, mostly palliative therapeutic options (van Zandwijk *et al*., 2013). MPM is highly related to asbestos exposure, and due to the long latency period and the widespread use of asbestos, the worldwide incidence is expected to further increase within the next decades (Linton *et al*., 2012; Robinson, 2012).

As classical malignant drivers such as mutated EGFR or Raf are uncommon in MPM, treatments specifically targeting these mutations are ineffective in this disease (Dubey *et al*., 2010; Garland *et al*., 2007; Govindan *et al*., 2005). Instead, genomic analysis has revealed genetic deletions in MPM largely in genes considered to be tumor suppressive, such as P16/CDKN2A and NF2 (Jaurand and Fleury‐Feith, 2005), which are not easily targetable. We and others have previously identified fibroblast growth factors (FGFs) and their receptors (FGFRs) as signaling molecules being overexpressed and driving malignant growth in MPM (Marek *et al*., 2014; Schelch *et al*., 2014). Hyperactivation of FGF receptors and downstream pathways has been associated with tumor progression, therapy resistance, and poor prognosis (Korc and Friesel, 2009). However, unlike, for instance, FGFR1 in non‐small‐cell lung cancer (NSCLC) and squamous cell lung cancer (Weiss *et al*., 2010) or FGF19 in hepatocellular carcinoma (HCC) (Sawey *et al*., 2011), recurrent gene amplification of FGFs or FGFRs was not detected in MPM and thus cannot account for the frequent overexpression of several FGF/R family members found in MPM (Marek *et al*., 2014).

MicroRNAs (miRNAs) are small noncoding RNAs post‐transcriptionally regulating gene expression via interaction with sites in the 3′UTRs of target mRNAs (He and Hannon, 2004). They are known to be important regulators of cell signaling proteins during cancer development and progression (Garzon *et al*., 2009). In particular, the miR‐15/16 microRNA family that targets numerous cancer‐relevant factors including the anti‐apoptotic protein Bcl‐2 has been shown to function as a tumor suppressor in various tumors (Bandi *et al*., 2009; Bhattacharya *et al*., 2009; Bonci *et al*., 2008), and we have shown that this is also the case in MPM (Reid *et al*., 2013).

Recently, miR‐16 was shown to directly interact with FGF2 in nasopharyngeal carcinoma (He *et al*., 2016), and several other members of the FGF/R family are predicted targets of miR‐15/16. Here, we experimentally validated the targeting of FGF1, FGF2, and FGF18 as well as FGFR1 and FGFR4 by the miR‐15/16 family in MPM, suggesting a potential mechanism contributing to the overexpression of the FGF axis in MPM. We demonstrate that miR‐15/16 replacement downregulates FGF/FGFRs and provide evidence that inhibition of FGF/FGFR‐dependent signals contributes to the growth suppressive effects of miR‐15/16 mimics. Furthermore, we identified a vicious cycle between FGF signaling and miR‐15/16 downregulation, driving malignant growth. Combined inhibition of two independent miR‐15/16 targets, FGFR1 and the Bcl‐2, resulted in synergistic growth inhibition, suggesting microRNA replacement as a novel therapy for FGFR‐dependent MPM and other FGFR‐dependent tumors.

## 2 Material and methods

### 2.1 Cell culture and cell lines

All cell lines were maintained in RPMI‐1640 or DMEM medium (Thermo Fisher, Waltham, MA, USA) containing 10% heat‐inactivated FBS in a humidified atmosphere of 5% CO_2_ at 37 °C. Cell lines were regularly checked for mycoplasma contamination (MycoFluor, Thermo Fisher) and STR profiling was used to confirm identity (GenePrint 10, Promega, Madison, WI, USA) once per year. All used cell lines, the histological MPM subtype they were derived from, their standard growth medium as well as their source are listed in Table S1.

### 2.2 MicroRNA mimics and siRNAs

MicroRNA mimics as well as validated negative control mimics (nc) were dissolved in ultrapure H_2_O and transfected as described below. For siRNAs, a nontargeting control siRNA (c‐81) was used. SiRNA specific for RRM1 served as control for transfection efficacy. All used microRNA mimics and siRNAs, their sequences/IDs and sources are listed in Table S2.

### 2.3 Cytokines and drugs

Recombinant FGF2 or FGFR/Bcl‐2 inhibitors were directly added to cells at the indicated concentrations. As control, equal amounts of solvent (PBS for FGF2, DMSO for all inhibitors) were added. Treatments, their targets and sources are summarized in Table S3.

### 2.4 Transfection with microRNA mimics and siRNAs

Reverse transfection with microRNA mimics or siRNAs was carried out as per the pipetting scheme in Table S4. First, mimics or siRNAs were diluted in serum‐free RPMI medium to the required concentrations. Lipofectamine (RNAiMAX, Thermo Fisher) was diluted 1 : 100 in serum‐free medium. After at least 5 minutes, appropriate volumes of lipofectamine and diluted mimic/siRNA were mixed and incubated for at least 20 minutes and up to 2 h at room temperature. Meanwhile, cells were harvested, counted, and diluted as required. Finally, the lipofectamine/RNA mix was added into each well and cells were added, gently mixed, and transferred to the incubator.

### 2.5 RNA isolation and RT‐qPCR

Cells were transfected in 6‐well plates (1.5 × 10^4^ cells per well) with microRNA mimics (5 nm) or siRNAs (10 nm) according to the scheme in Table S4. After the indicated time, RNA was isolated using TRIzol (Thermo Fisher) according to the manufacturer's instructions and dissolved in ultrapure H_2_O. Concentration and purity of the RNA were measured on a nanophotometer (Implen, Munich, Germany). For the analysis of target genes, 500 ng RNA was reverse‐transcribed with Superscript III reverse transcriptase (Thermo Fisher). To measure the expression of microRNAs, 50 ng RNA was reverse‐transcribed using MultiScribe reverse transcriptase (Thermo Fisher) and stem‐loop RT primers specific for the microRNA of interest. SYBR green‐ or TaqMan‐based real‐time quantitative PCR was performed according to the manufacturer's instructions on an ABI‐7500 (Applied Biosystems, Foster City, CA, USA) or ViiA7 (Thermo Fisher) thermocycler. 18S was used as a reference for mRNA, and RNU6B and miR‐191 as references for microRNA expression, respectively. All used TaqMan microRNA assays are listed in Table S5. Primers and sequences for SYBR green‐based PCR are in Table S6. Relative levels of mRNAs or microRNAs were calculated as previously described (Reid *et al*., 2013) using the 2^−ΔΔCq^ method (Livak and Schmittgen, 2001).

### 2.6 Luciferase reporter assay

Fragments of the 3′UTRs of FGF1, FGF2, FGF5, FGF18, FGFR1, and FGFR4 containing binding sites for miR‐15a, miR‐15b, and miR‐16 were cloned from SPC212 cDNA. Total RNA was reverse‐transcribed (MMLV RT kit, Promega) and fragments were amplified using AmpliTaq Gold 360 (Promega) with specific forward and reverse primers (Table S7). PCR products were first cloned into the TOPO TA vector (Thermo Fisher) and then subcloned into the pSiCheck2 plasmid (Promega). The mutated FGFR1 constructs were generated by site‐directed mutagenesis. Briefly, specific, completely overlapping primers harboring a mutation in the microRNA binding site were designed (Table S7) and a PCR was carried out using PfuUltra II Fusion HS DNA Polymerase (Agilent, Santa Clara, CA, USA) followed by DpnI (NEB, Ipswich, MA, USA) digest. Sequences were confirmed by Sanger sequencing carried out at the Ramaciotti Centre (UNSW, Sydney). The resulting reporter constructs (1 μg), together with microRNA mimics or controls (5 nm), were used to transfect 2 × 10^5^ cells in 6‐well plates. A dual luciferase assay (Promega) was carried out as per the manufacturer's protocol 48 h after transfection.

### 2.7 Protein isolation and western blot

For protein analysis, 4 × 10^5^ cells in T25 flasks were transfected with 2.5 nm microRNA mimics. After 96 h, protein was isolated in lysis buffer (150 mm NaCl, 50 mm HEPES, 10% glycerol, 1 mm EDTA, 0.5 mm Na_3_VO_4_, 10 mm NaF, 1% Triton X‐100, and 1.5 mm MgCl_2_) and concentration was measured using a Bradford protein assay (Bio‐Rad, Hercules, CA, USA). 20 μg of protein per lane was separated by SDS/PAGE (precast, NuSep) and blotted onto PVDF membranes. Immunodetection was performed using antibodies against FGFR1 (D8E4 XP, #9740, Cell Signaling; 1 : 1000) and FGFR4 (H‐121, sc‐9006, Santa Cruz, Dallas, TX, USA; 1 : 1000). Beta‐actin (#A5441, Sigma, St. Louis, MO, USA; 1 : 10 000) served as loading control.

### 2.8 Growth inhibition assay

Triplicate of 2.5 × 10^3^ MPM cells were transfected with microRNA mimics, siRNAs, or various combinations as indicated and seeded in a 96‐well plate with a total volume of 120 μL per well. Usually, mimics were used at final concentrations of 0.2 nm, 1 nm, and 5 nm and siRNAs at 10 nm. In case of additional treatment with cytokines or drugs, 80 μL of medium containing the compound at the required concentrations or vehicle was added to a total volume of 200 μL per well. To test the effects of drugs alone, 2 × 10^3^ cells per 96‐well in a volume of 100 μL medium were seeded in triplicate. On the next day, cells were treated with 100 μL of medium containing drugs single or in combination as indicated. At the indicated time points, the medium was discarded and plates were frozen at −80 °C. For analysis, plates were thawed and 200 μL lysis buffer (10 mm Tris/HCl pH = 8, 2.5 mm EDTA, 0.1% Triton X‐100) containing SYBR green (10 000×, Thermo Fisher, 1 : 8000) was added. Plates were incubated overnight at 4 °C in the dark. Fluorescence was measured at 485/520 nm on a FLUOstar OPTIMA microplate reader (BMG Labtech, Offenburg, Germany).

### 2.9 MTT assay

Triplicate of 2 × 10^3^ cells in 100 μL medium were seeded into a 96‐well plate. On the next day, 100 μL of medium containing treatments was added. DMSO was used as solvent control. After 72 h, cell viability was measured by MTT assay according to the manufacturers' protocol (EZ4U, Biomedica, Vienna, Austria).

### 2.10 Colony formation assay

Cells (2.5 × 10^3^) were transfected with mimics in 96‐well plates as described above. On the next day, cells from each well were harvested and half transferred into each of two wells of a 6‐well plate, resulting in approximately 1000–1500 cells per well, and returned to the incubator. After 7–14 days, cells were fixed with methanol/acetic acid (3 : 1) and clones were stained with crystal violet and air‐dried and pictures were taken. For quantification, clones were destained in 2% SDS, and absorbance was read at 562 nm.

### 2.11 Statistical analysis

If not stated otherwise, all data are presented as means ± SEM of at least three independent experiments performed in triplicate. Statistical significance between control and treatment groups was calculated using Prism7 (GraphPad, San Diego, CA, USA) using one‐way ANOVA with Dunnett's multiple comparison test for comparison of multiple groups. Pearson's correlation was used to investigate correlations between the effect of microRNAs re‐expression on cell growth and sensitivity to pharmacological FGFR inhibition. A *P*‐value <0.05 was considered statistically significant. AUCs were calculated with Prism7. Combination indices (CI values) were calculated using the compusyn software (CompuSyn Inc., Paramus, NJ, USA). Predicted values (PV) for additive effects represent the arithmetic products of the % viability of each single treatment.

## 3 Results

### 3.1 Downregulation of the microRNA‐15/16 family corresponds with an upregulation of the FGF axis in MPM cell lines

Recently, our work has shown hyperactivation of FGF signals due to overexpression of FGFR1, FGF2, and FGF18 in MPM (Schelch *et al*., 2014). As there are no gene amplifications or mutations reported, we reasoned that microRNAs might play a role in the aberrant FGF/R expression. We used target prediction (TargetScanHuman 7.1, Agarwal *et al*., 2015) and found that the tumor‐suppressive miR‐15/16 microRNA family, which is frequently downregulated in tumors including MPM (Reid *et al*., 2013), is predicted to target several members of the FGF axis.

**Figure 1:**
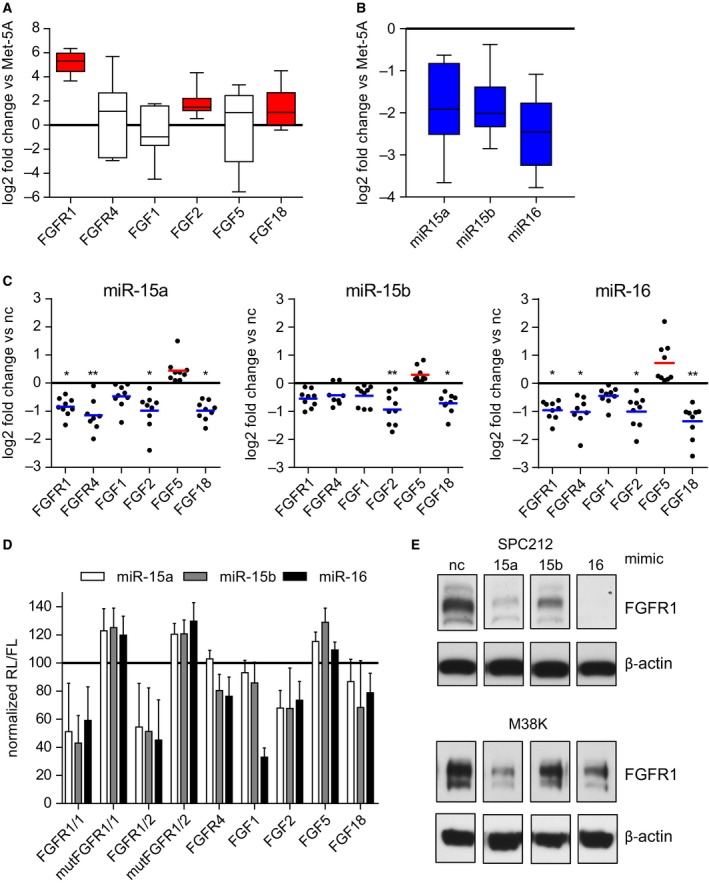
The microRNAs 15a, 15b, and 16 are downregulated in mesothelioma cells and target several members of the FGF family. Expression of (A) several members of the FGF axis and (B) the microRNA‐15/16 family compared to MeT‐5A, analyzed by RT‐qPCR. (C) Cells were transfected with microRNA mimics (5 nm) and target gene expression was checked after 24 h via RT‐qPCR and compared with noncoding (nc) control mimics. Each dot represents one cell line and is depicted as the mean of three independent experiments. (D) Normalized percentage of luciferase activity (renilla/firefly ratio, RL/FL) 48 h after transfection with microRNA or control mimics (5 nm) in SPC212 cells. (E) Immunoblot analysis of FGFR1 expression 96 h after transfection with microRNA or control mimics (2.5 nm). *P < 0.05, **P < 0.01, ***P < 0.001; otherwise, *P* > 0.05.

To investigate the relationship between the miR‐15/16 family and the FGF axis, we first measured the expression of miR‐15a, miR‐15b, and miR‐16 as well as several members of the FGF/FGFR family in a panel of seven MPM cell lines (plus five drug‐resistant derivatives) via RT‐qPCR. MeT‐5A, a nonmalignant transformed mesothelial cell line, was used as a reference. We found a prominent overexpression of FGFR1, FGF2, and FGF18 (Fig. 1A) as well as reduced expression of the microRNAs miR‐15a, 15b, and 16 in the MPM cell lines compared with MeT‐5A (Fig. 1B).

### 3.2 The miR‐15/16 family targets the FGF axis in MPM

In order to directly assess the impact of the miR‐15/16 family members on FGF/FGFR expression in MPM cells and to validate the prediction of FGF/FGFRs as miR‐15/16 targets in MPM, we reintroduced each of the three miRNAs into the MPM cell lines via transfection with mimics and measured target gene expression via RT‐qPCR. All three miRNA mimics led to downregulation of the expression of FGFR1 and FGFR4, which are both predicted targets of the respective miRNAs. Among the ligands, FGF1, FGF2, and FGF18 are predicted targets and all three were also reduced upon mimic expression (Fig. 1C). FGF5 is not a target of the miR‐15/16 family and, as expected, was not downregulated. Luciferase reporter assays confirmed the direct interaction between miR‐15a, 15b, and 16 and their predicted target sites in the 3′UTR of FGFR1 (FGFR1/1, FGFR1/2), FGFR4, FGF1, FGF2, and FGF18, but no interaction with FGF5 or mutated target sites of FGFR1/1 or FGFR1/2 was found. (Fig. 1D). We also observed a downregulation of FGFR1 on the protein level after transfection with miRNA mimics (Fig. 1E). This was more pronounced with miR‐15a and miR‐16, reflecting the changes in the mRNA level. These findings establish several FGF/FGFR members as experimentally validated targets of the miRNA 15/16 family in mesothelioma.

### 3.3 Growth repression by miRNA mimics correlates with sensitivity to FGFR1 inhibition in MPM cells

**Figure 2:**
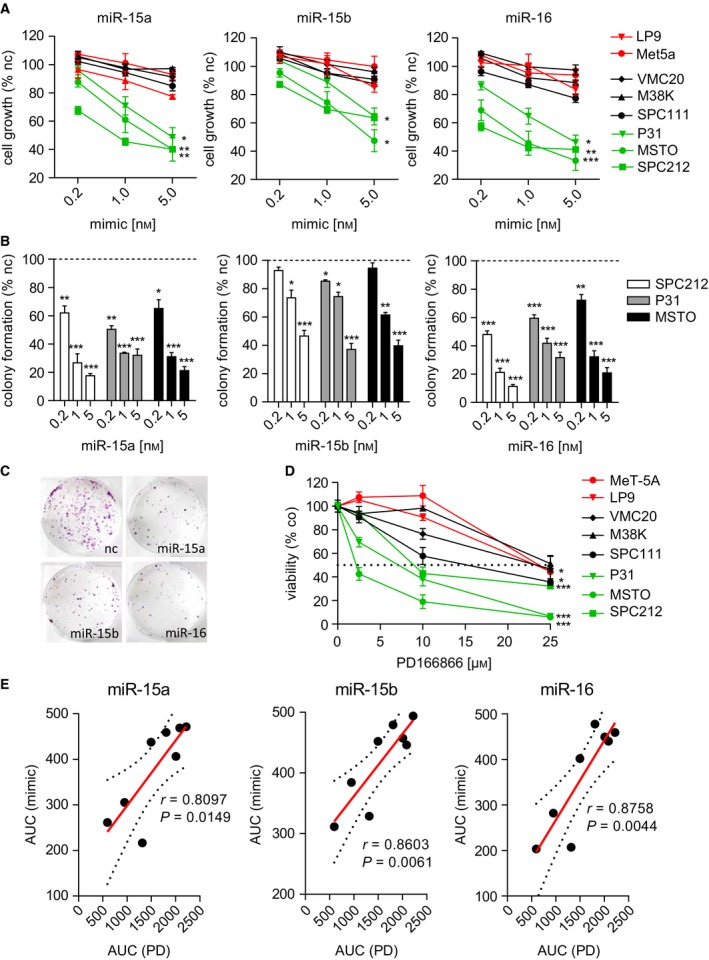
Growth repression by microRNA mimics correlates with sensitivity to FGFR1 inhibition. (A) Growth inhibition determined by SYBR green staining 72 h after transfection with mimics compared with noncoding control (nc). (B) Quantification of colony formation assays of the three sensitive cell lines after transfection with mimics as indicated. (C) Representative colony formation pictures of MSTO cells transfected with 5 nm microRNA or control mimics as indicated. (D) Dose–response curves of MPM cell lines 72 h after treatment with PD166866 or DMSO (co), determined by MTT assay. (E) Growth inhibition shown as area under curve (AUC) of mimics (5 nm) in correlation (Pearson) effects of PD166866, calculated from MTT dose–response curves in E. Each dot represents one cell line. *P < 0.05, **P < 0.01, ***P < 0.001; otherwise, *P* > 0.05.

In line with our previous findings, transfection with miRNA mimics led to a dose‐dependent growth repression of MPM cells (Fig. 2A). In agreement with the effects on target gene expression, miR‐15b also had the weakest impact on MPM cell growth. The strongest effect was seen in MSTO, SPC212, and P31 cells, while VMC20, M38K, SPC111 as well as the control mesothelial cell lines MeT‐5A and LP9 were only moderately affected. Restoration of miR‐15/16 also dose dependently reduced the ability to form colonies in MPM cells when plated at low density (Figs 2B and C, S1).

We previously reported that FGFR1 inhibition reduces MPM tumor growth *in vitro* and *in vivo* (Schelch *et al*., 2014). In our panel of MPM cell lines, MTT assays using the small‐molecule‐specific FGFR1 inhibitor PD166866 showed a dose‐dependent decrease in cell viability reflecting the effects seen by miRNA mimics (Fig. 2D). There was a significant correlation (Pearson) between the effectiveness of growth repression by mimic expression and sensitivity to PD166866 (Fig. 2E). The fact that those cell lines which are sensitive to miR‐15/16 are also more responsive to FGFR1 inhibition suggests that blockade of FGF/FGFR‐dependent growth/survival signals is an important player in growth regulation by this microRNA family.

### 3.4 Stimulation with FGF2 can rescue mimic‐induced growth repression of MPM cells at early but not late time points

**Figure 3:**
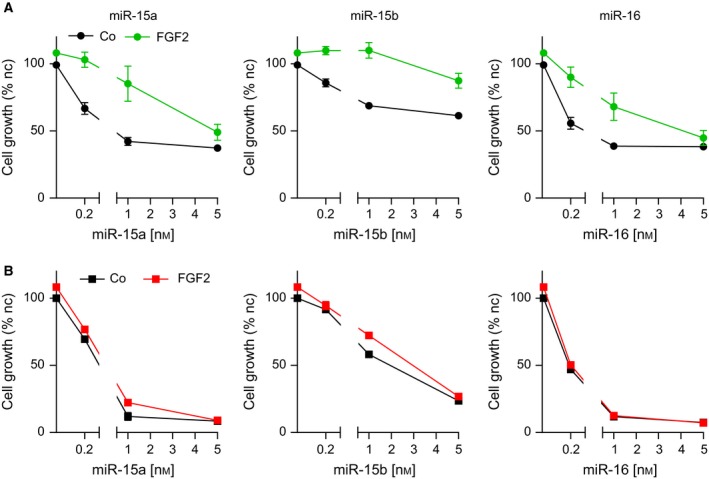
Stimulation with FGF2 reduces mimic‐induced growth inhibition of MPM cells at early but not late time points. Growth inhibition assay of SPC212 cells after transfection with mimics at indicated concentrations in combination with FGF2 treatment (10 ng·mL^−1^) (A) 24 h after mimic transfection or (B) 96 h after transfection. For the latter, each well was split 1 : 3 on the day before treatment to avoid complete confluence. Assays were quantified 72 h after FGF2 treatment. *P < 0.05, **P < 0.01, ***P < 0.001; otherwise, *P* > 0.05.

We reasoned that in MPM cells dependent on FGF signals for cell growth and survival, growth suppression upon mimic‐induced downregulation of FGFs would be prevented by stimulation with exogenous FGF2 as long as FGFRs are sufficiently expressed. Therefore, to further characterize the interaction between miR‐15/16 and the FGF axis, we stimulated the cells at two different time points: 24 and 96 h after transfection. Indeed, FGF2 treatment 24 h after transfection could reduce/prevent the growth inhibition effects caused by mimics (Figs 3A and S2). In contrast, when added 96 h after transfection, FGF2 had no effect on cell growth (Figs 3B and S2). This is most likely explained by reduced FGFR1 protein at this late time point as shown in Fig. 1E.

### 3.5 MicroRNAs targeting the FGF axis are regulated by FGFR‐mediated signals in a vicious cycle

**Figure 4:**
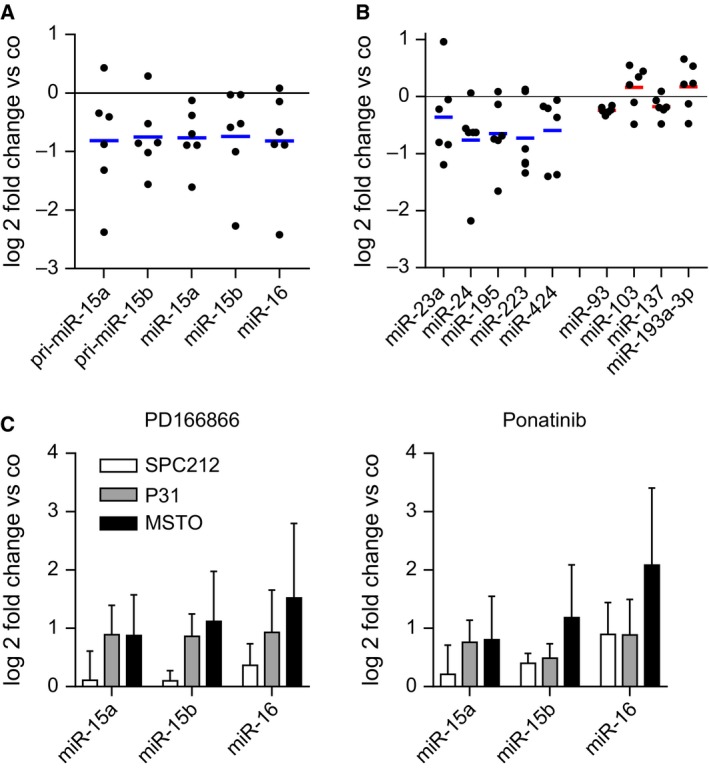
Inhibition or stimulation of FGF signals regulates the microRNA‐15/16 family. (A) Expression of the miR‐15a, miR‐15b, miR‐16, and their respective pri‐miRs or (B) miR‐23a, miR‐24, miR‐195, miR‐223, and miR‐424 as well as miR‐93, miR‐103, miR‐137, and miR‐193a‐3p after treatment with FGF2 (10 ng·mL^−1^) for 24 h compared with control cells was determined by qPCR. Each dot represents a cell line and is depicted as the mean of three experiments performed in duplicate. (C) MicroRNA expression determined by qPCR after 24 h treatment with PD166866 (10 μm) or ponatinib (500 nm) and respective vehicle controls (Co). *P < 0.05, **P < 0.01, ***P < 0.001; otherwise, *P* > 0.05.

Our data suggest that one of the functions of the miR‐15/16 family is to keep FGF signals in check. As regulation of growth signals often involves feedback loops to maintain homeostasis, we next investigated whether activated FGF signals would in turn enhance microRNA expression. We determined the expression of miR‐15a, miR‐15b, miR‐16, and their respective precursor pri‐miRs via Taqman‐based RT‐qPCR after 24‐h stimulation with recombinant FGF2 and found further downregulation of miR‐15/16 in the majority of cell lines (Fig. 4A, Table S8). However, there was no significant correlation between expression changes and sensitivity to mimics/FGFR inhibition (data not shown). In addition to miR‐15a, ‐15b, and ‐16, miR‐195, and miR‐424—additional members of this family—as well other microRNAs predicted to target various members of the FGF axis—miR‐23a, miR‐24, and miR‐223—were also downregulated upon FGF pathway activation. In contrast, miR‐93, miR‐103, miR‐137, and miR‐193a‐3p, which are not involved in the regulation of the FGF axis, remained largely unchanged (Fig. 4B, Table S8). These data suggest that a feed‐forward cycle is operational in the mutual regulation between the miR‐15/16 family and the FGF axis in MPM cells. Once triggered, this feed‐forward loop could result in a vicious cycle contributing to enhanced cell growth in MPM. This model would suggest that, conversely, inhibition of FGF signals could partially rescue miR‐15/16 expression. Therefore, we treated the three sensitive cell lines with the FGFR1 inhibitor PD166866 as well as the multikinase inhibitor ponatinib, which inhibits FGFR1‐4, Abl, PDGFRA, and Src, and assessed the expression of miR‐15/16. Indeed, after 48 h, we observed with both treatments an upregulation of miR‐15a, ‐15b, and ‐16 (Fig. 4C).

### 3.6 Competition between microRNA restoration and FGFR inhibition confirms interaction between miR‐15/16 and the FGF axis

**Figure 5:**
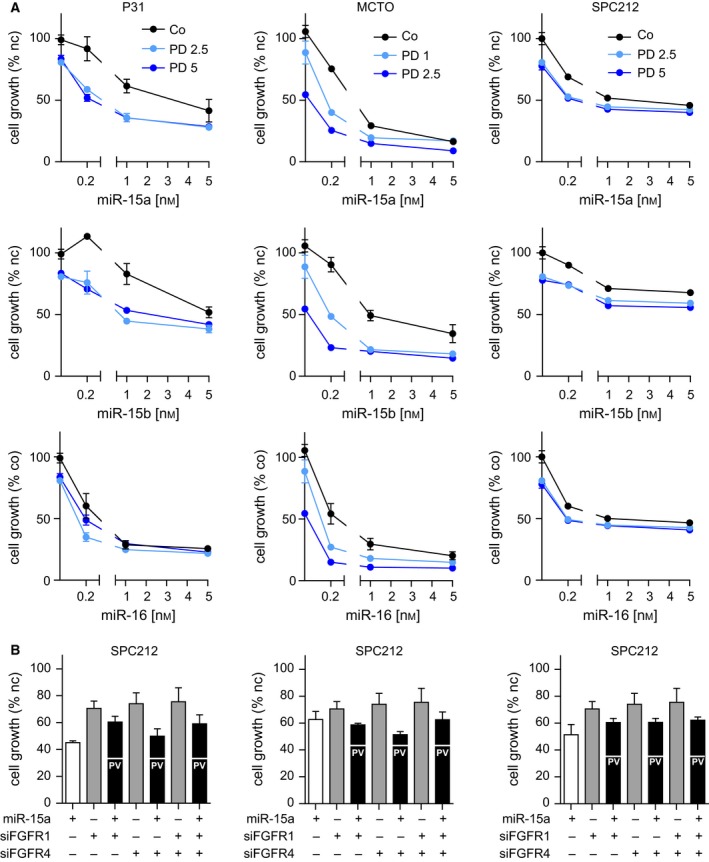
Combination of microRNA restoration and FGFR inhibition or knockdown shows effects of target competition. (A) Growth inhibition assay of MPM cells after transfection with microRNA or noncoding control mimics in combination with PD166866. Treatment was for 72 h with the indicated concentrations (1 μm, 2.5 μm, or 5 μm). DMSO was used as solvent control. (B) Growth inhibition assay 72 h after cells were transfected with mimics (5 nm) and/or siRNAs (10 nm) and/or respective controls (nc). Predicted values of additive interaction (PV) are indicated as white lines within the black combination bars. *P < 0.05, **P < 0.01, ***P < 0.001; otherwise, *P* > 0.05.

As our data suggest that mutual regulation between miR‐15/16 and FGF signals drives enhanced growth of MPM cells, interference with either one or both mechanisms could prove therapeutically beneficial. Indeed, we have previously demonstrated that restoration of miR‐16 is feasible in MPM models and results in growth suppression *in vitro* and *in vivo* (Reid *et al*., 2013) and several groups including our own have established FGFR inhibition as viable anti‐MPM strategy (Blackwell *et al*., 2016; Marek *et al*., 2014; Schelch *et al*., 2014). Thus, we tested the effect of combining microRNA replacement with pharmacological FGFR inhibition. The combination of mimic expression with the FGFR1 inhibitor PD166866 reached a higher total growth inhibition compared with each treatment alone. However, with higher doses of mimics, the combinations showed reduced effects compared with the single treatments, indicating target competition (Fig. 5A). Comparable results, showing effects weaker than the predicted value (PV, indicated as white ticks within the black bars) of additive interactions, were obtained when we combined miRNA mimics with siRNA against FGFR1, FGFR4, or a combination of both (Figs 5B, S3 and S4).

### 3.7 Combined inhibition of the miR‐15/16 targets FGFR1 and Bcl‐2 has synergistic effects in MPM cells

**Figure 6:**
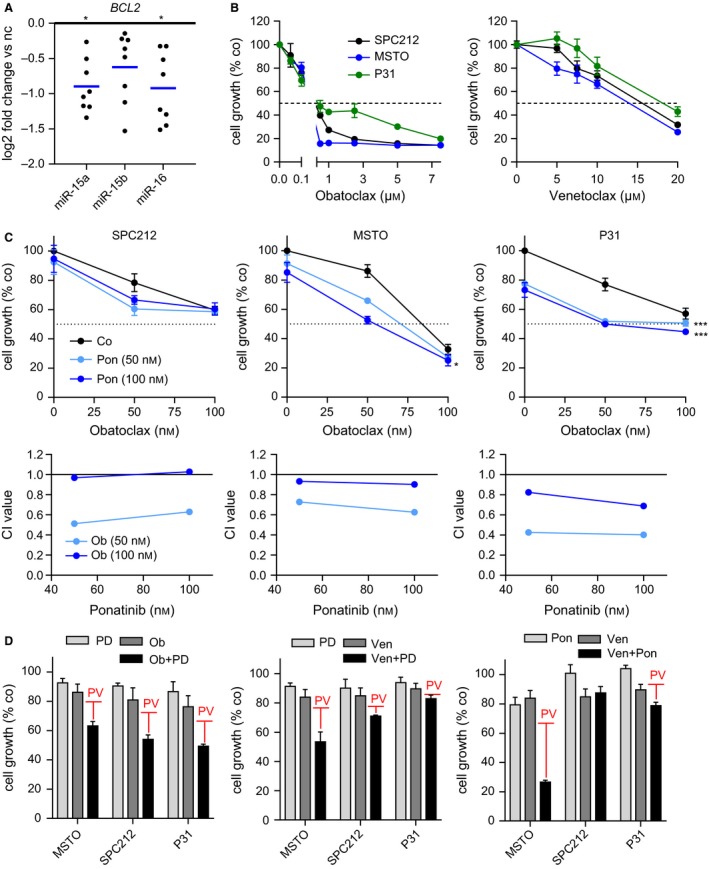
Pharmacological inhibition of the FGF axis in combination with Bcl‐2 has synergistic effects in MPM. (A) Expression analysis of *BCL2* via qPCR 24 h after transfection with microRNA mimics (5 μm) compared with noncoding (nc) control mimics. (B) Growth inhibition assays using obatoclax and venetoclax as single agents at indicated concentrations for 72 h. DMSO was used as solvent control. (C) Growth inhibition of MPM cells after treatment with ponatinib (Pon, 50 nm, 100 nm) in combination with obatoclax (Ob, 50 nm, 100 nm) for 72 h. DMSO was used as solvent control. Combination indices (CI values) were calculated using CompuSyn. (D) Growth inhibition of PD166866 (PD, 1 μm) or ponatinib (Pon, 100 nm) in combination with obatoclax (Ob, 50 nm) or venetoclax (Ven, 10 μm) for 72 h. The red bars represent the predicted value of additive interaction (PV) of the two respective single treatments. *P < 0.05, **P < 0.01, ***P < 0.001; otherwise, *P* > 0.05.

We reasoned that inhibition of two independent targets of miR‐15/16 might avoid the target competition seen in Fig. 5. A well‐established target of the miR‐15/16 family is the anti‐apoptotic protein Bcl‐2, for which both the clinically relevant specific inhibitor venetoclax and the broadly active Bcl‐2 family inhibitor obatoclax are available. First, we confirmed the downregulation of Bcl‐2 upon transfection with microRNA mimics in our panel of cell lines via RT‐qPCR. As expected, Bcl‐2 levels were reduced in all cell lines (Fig. 6A). Therefore, we next tested the FGFR1 inhibitor PD166866 as well as the multikinase inhibitor ponatinib in combination with venetoclax and obatoclax. Used as single agents, obatoclax was highly effective in the nanomolar range; venetoclax, however, required much higher concentrations to inhibit growth in MPM cells (Fig. 6B). Importantly, in combination with low‐dose FGFR inhibitors, we found enhanced effects of both obatoclax and venetoclax. The combination of obatoclax and ponatinib showed synergistic activity in all three cell lines tested, with combination indices (CI) below 1, especially at the lower concentrations (Fig. 6C). In the other combination settings, where calculation of CI values was not possible, effects stronger than the predicted value (PV, indicated as red bars) of additive interactions were observed at concentrations of 1 μm PD166866 or 100 nm ponatinib in combination with 50 nm obatoclax or 10 μm venetoclax (Figs 6C and S5).

## 4 Discussion

The translation of specific molecularly targeted approaches into clinical practice, often in combination with conventional chemo‐ or radiotherapy, has become a promising strategy, which is currently improving the treatment for various other cancers (Iams *et al*., 2017; Lemjabbar‐Alaoui *et al*., 2015). However, MPM remains an exception. Apart from a modest increase in median survival by the addition of bevacizumab to the standard pemetrexed/platinum combination, this asbestos‐related cancer (Zalcman *et al*., 2015), with increasing worldwide incidence, continues to be renowned for its treatment resistance. Therefore, novel insight into the molecular basis of treatment resistance and more effective treatment strategies are urgently needed. Our previous work characterized the FGF axis as a potent malignant driver in MPM, and we also described the tumor‐suppressive role of the miR‐15/16 family, which is frequently downregulated in MPM (Reid *et al*., 2013; Schelch *et al*., 2014).

So far, the tumor suppressor function of miR‐15/16, which targets important drivers of malignant growth such as FGF2 and the anti‐apoptotic protein Bcl‐2 (He *et al*., 2016; Willimott and Wagner, 2010), has been well established in various tumor types including prostate, lung, ovarian, and MPM (Bandi *et al*., 2009; Bhattacharya *et al*., 2009; Bonci *et al*., 2008; Reid *et al*., 2013). We demonstrate here that several members of the FGF axis (FGFR1, FGFR4, FGF1, FGF2, and FGF18) are prominent miR‐15/16 targets, downregulated in MPM cells upon microRNA restoration via mimics.

The loss of post‐transcriptional control of the FGF axis due to downregulation of microRNAs is a potential mechanism underlying the high expression of the FGF axis in tumors such as MPM, which do not show gene amplifications or mutations (Marek *et al*., 2014). However, a lack of a significant correlation between basal FGF/R levels and miR‐15/16 expression suggests that the levels of this microRNA family contribute to, but do not completely control, regulation of FGF/R expression. Besides miR‐15/16, other microRNAs have also been shown to impact on FGF signals in malignant and nonmalignant diseases, including miR‐99a, miR‐214, or miR‐216 (Jiang *et al*., 2014; Wang *et al*., 2008, 2013). Interestingly, miR‐99a was found at lower levels in MPM samples compared with normal pleura (Andersen *et al*., 2014) and differentially expressed between epithelioid and sarcomatoid tumors (Pass *et al*., 2010). Similarly, miR‐214 was shown to be downregulated in MPM samples compared with controls in two independent studies (Amatya *et al*., 2016; Balatti *et al*., 2011). In addition, we found that FGF signaling suppressed the expression of miR‐23a and miR‐223, both of which are altered in MPM and predicted to target components of the FGF axis (Birnie *et al*., 2015; Cheng *et al*., 2013).

Previously, high levels of both amplified and nonamplified FGFRs have been linked to response to FGFR inhibition in various cancers (Goke *et al*., 2015; Nakanishi *et al*., 2014; Wynes *et al*., 2014; Zhang *et al*., 2014). In accordance with this, we have recently shown that nonmalignant mesothelial cells express much lower levels of FGF/R and are more resistant to FGFR inhibition than MPM cells (Schelch *et al*., 2014). However, among the MPM cell lines tested, neither FGF/R nor miR‐15/16 basal expression correlated with sensitivity to FGFR inhibition or mimics. Also, comparable to MeT‐5A, some MPM cell lines did not respond to growth inhibition. This suggests that the actual signals which trigger cell proliferation and survival and therefore the extent of dependency on the FGF axis are controlled by a combination of multiple factors including transcriptional and translational regulation and other effectors acting downstream of the FGF receptors. MicroRNAs targeting specific components of the FGF family represent one part of this regulatory system, which is supported by effects of target competition when we combined microRNA mimics with FGFR inhibitors. Also, it is well known that the FGF axis is part of a complex signaling network, which interacts with numerous other pathways and is influenced by various feedback and feed‐forward loops (Ornitz and Itoh, 2001). We have uncovered a vicious cycle in MPM where FGF signaling is capable of downregulating the miR‐15/16 family and other FGF/R‐targeting microRNAs, thereby further driving their growth‐promoting effects by inhibiting their own microRNA‐mediated post‐transcriptional regulation.

The involvement of the same tumor suppressor microRNAs in the regulation of multiple cancer‐related signaling pathways highlights the potential of microRNA mimics as novel cancer treatment approach. In addition, it is unlikely that microRNA‐based therapy is negatively affected by mutation‐induced resistance usually occurring with tyrosine kinase inhibitors. MicroRNA replacement has been evaluated in preclinical and clinical studies (Bader, 2012; Liang *et al*., 2016; Reid *et al*., 2013; Takeshita *et al*., 2010). In MPM, a miR‐16‐based mimic has shown early signs of clinical activity (Kao *et al*., 2015). As the underexpression/loss of members of the miR‐15/16 family is not a unique characteristic of MPM and considering the important role of the FGF axis in tumor development and progression, it is more than likely that the deregulation of FGF signals also contributes to the tumor‐promoting effects of miR‐15/16 loss in other tumor types. Therefore, the miR‐16 suppletion approach needs further investigation for tumors characterized with mutated or dysregulated FGF signaling.

We demonstrate here that selecting microRNA target proteins which (a) have a well‐documented role in driving malignant growth and (b) can be targeted by pharmacological inhibition represents a rational approach for combination treatment in MPM. This approach enabled us to identify that FGFR1 and Bcl‐2 represent novel synergistic treatment targets in MPM. Multiple members of the miR‐15/16 family may be targeted with microRNA mimics, whereas FGFRs and Bcl‐2 have the advantage of being druggable. FGFR inhibition has shown promising effects in various preclinical studies (Dey *et al*., 2010; Maruyama‐Takahashi *et al*., 2008; Metzner *et al*., 2011; Pattarozzi *et al*., 2017; Qing *et al*., 2009; Schelch *et al*., 2014) and is currently being tested in patients with solid tumors, especially in those with known FGFR overexpression or genomic alterations (Katoh and Nakagama, 2014). In mesothelioma, a phase lll study using the multi‐RTK inhibitor nintedanib (NCT01907100) and a phase lb trial using the FGFR ligand trap GSK3052230 in combination with first‐line chemotherapy (NCT01868022) are ongoing. Similarly, inhibition of Bcl‐2 also showed promising effects in various cancers including MPM (Hoda *et al*., 2016; Li *et al*., 2008; Schwartz *et al*., 2016; Xiong *et al*., 2016). Other combined treatment approaches have been tested in MPM (Kanteti *et al*., 2014; Ou *et al*., 2016), and a trial of FAK and PD1 inhibition (NCT02758587) is ongoing. With the lack of targetable mutations in MPM, novel treatments focusing on gene expression and genomic alterations are gaining increasing attention. For example, a trial of the EZH2 inhibitor in the context of BAP1 loss‐of‐function mutation (NCT02860286) is currently recruiting. Similarly, our characterization of the impact of microRNA expression changes on MPM biology led us to identify the synergistic effect of combining inhibitors of FGFR1 and Bcl‐2, two independent targets of the miR‐15/16 pathway. With the limited progress in MPM treatment over the last decade, continuing use of alternative approaches for the identification of novel MPM treatment targets will be needed to improve the outlook for patients.

In conclusion, our study suggests that loss of the miR‐15/16 family plays a role in the overexpression of the FGF axis in MPM. We identified a vicious cycle of malignant growth between FGF signals and miR‐15/16 and show that cells which are more sensitive to FGFR inhibition also respond better to microRNA mimics, providing evidence for microRNA replacement as an alternative therapeutic approach in MPM and other tumors harboring FGF/R mutations or amplifications. Furthermore, we demonstrate that combined inhibition of two miR‐15/16 targets, FGFR1 and Bcl‐2, has synergistic effects on tumor cell growth encouraging further consideration as novel combination strategy in MPM.

## Author contributions

GR, MG, NvZ, and KS designed the study. KS, MBK, YYC, and MW conducted experiments and performed data analysis. KS, MBK, NvZ, and GR wrote the manuscript. All authors read and approved the final manuscript.

## Supporting information


**Fig. S1.** Representative pictures of MSTO cells in a colony formation assay after transfection with microRNA mimics or non‐coding control.
**Fig. S2.** Growth inhibition assays of P31 and MSTO cells after transfection with mimics in combination with FGF2 treatment.
**Fig. S3.** Target gene and protein expression analysis after transfection with siRNAs via qPCR or western blot.
**Fig. S4.** Growth inhibition assays of P31 and MSTO cells 72 h after transfection with microRNA mimics and/or siRNAs or respective controls.
**Fig. S5.** Growth inhibition assays of SPC212, MSTO and P31 cells 72 h after treatment with PD166866 (PD) or ponatinib (Pon) in combination with obatoclax or venetoclax, or respective controls, at the indicated doses.

**Table S1.** List of used cell lines, the histological MPM subtype they were derived from, standard growth media and sources.
**Table S2.** List of mimics and siRNA, their sequences/IDs and sources.
**Table S3.** List of cytokines and drugs, their targets and sources.
**Table S4.** Transfection schemes for different assay formats.
**Table S5.** List of primers and probes used in TaqMan‐based reverse transcription and qPCR.
**Table S6.** Primers and their sequences used for SYBR green‐based qPCR.
**Table S7.** Primers and their sequences used for Luciferase reporter assays.
**Table S8.** Fold change, standard deviation and *P*‐values for microRNAs of individual cell lines after treatment with FGF2.
