# Supplementary material for: A link between the fibroblast growth factor axis and the miR‐16 family reveals potential new treatment combinations in mesothelioma
Source: Mol Oncol. 2017 Nov 18;12(1):58–73. doi: 10.1002/1878-0261.12150 (PMC5748487; doi:10.1002/1878-0261.12150)
Supplement: Supplementary file 1 — Fig. S1. Representative pictures of MSTO cells in a colony formation assay after transfection with microRNA mimics or non‐coding control. Fig. S2. Growth inhibition assays of P31 and MSTO cells after transfection with mimics in combination with FGF2 treatment. Fig. S3. Target gene and protein expression analysis after transfection with siRNAs via qPCR or western blot. Fig. S4. Growth inhibition assays of P31 and MSTO cells 72 h after transfection with microRNA mimics and/or siRNAs or respective controls. Fig. S5. Growth inhibition assays of SPC212, MSTO and P31 cells 72 h after treatment with PD166866 (PD) or ponatinib (Pon) in combination with obatoclax or venetoclax, or respective controls, at the indicated doses. [file MOL2-12-58-s001.pdf]

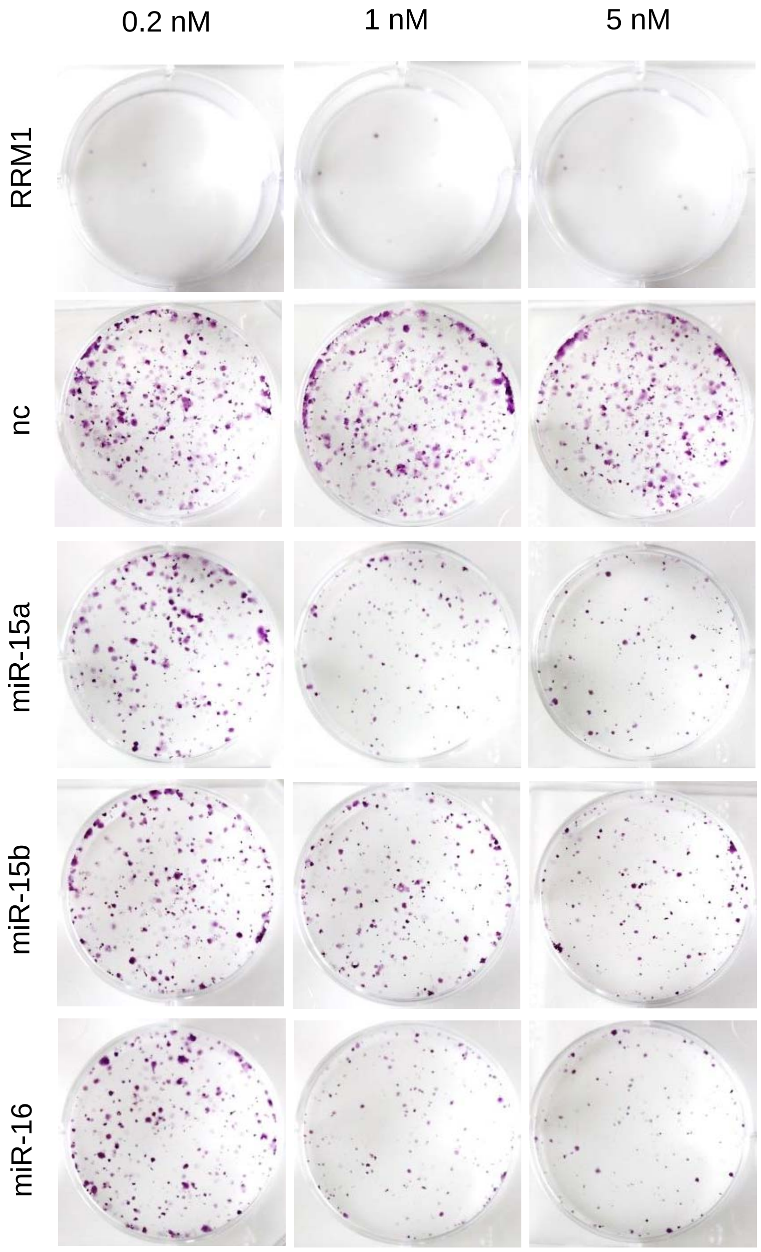

Fig. S1: Representative pictures of MSTO cells in a colony formation after transfection with microRNA mimics or non coding control (nc) with the indicated concentrations. RRM1 siRNA was used as transfection control.

Schelch et al. Fig. S1

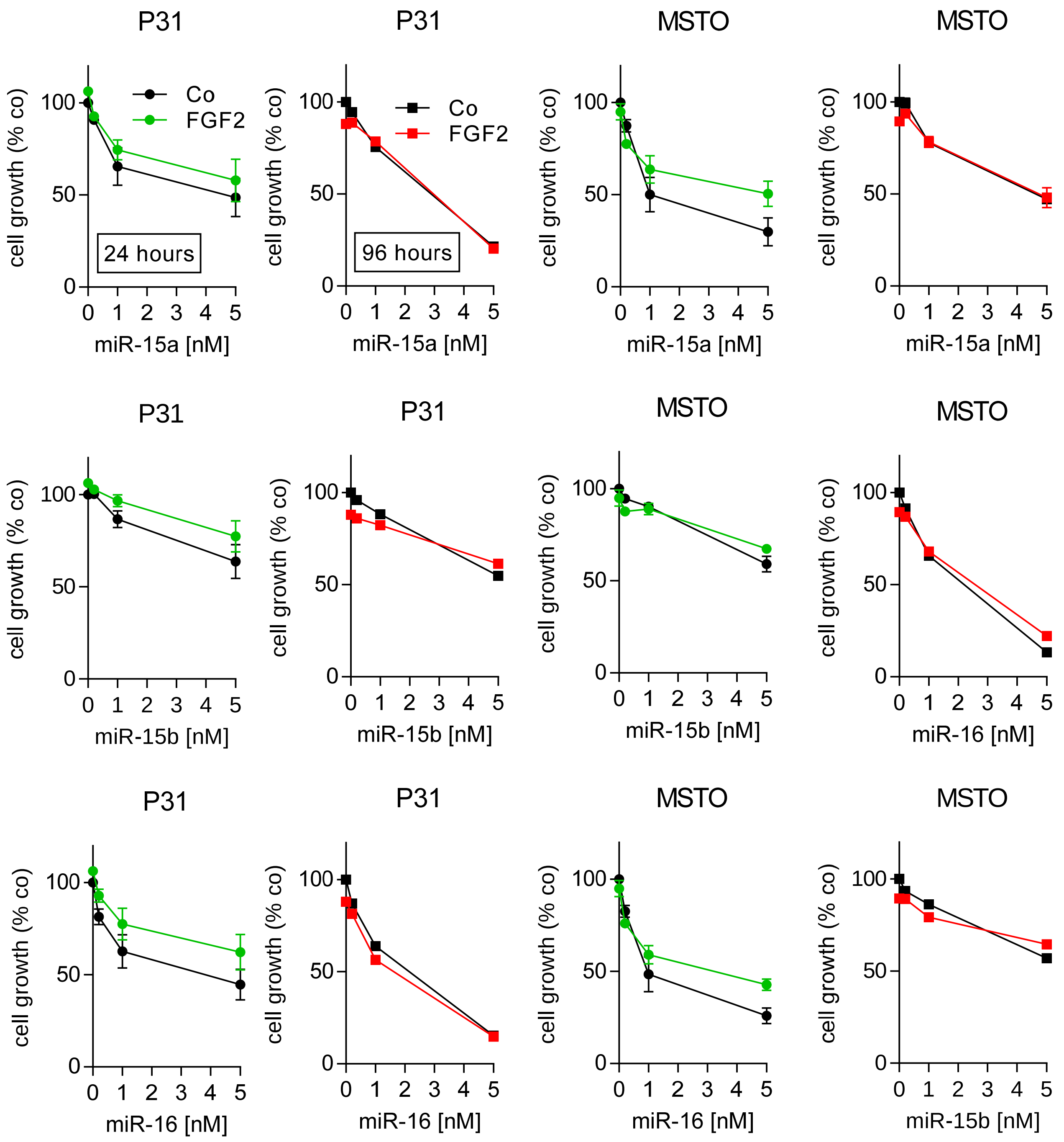

Fig. S2: Growth inhibition assays of P31 and MSTO cells after transfection with mimics in combination with FGF2 treatment (10 ng/ml) 24 h after mimic transfection (green). In case of FGF2 treatment 96 h after transfection (red), each well was split 1:3 on the day before treatment to avoid complete confluence. Assays were stopped 72 h after FGF2 treatment.

Schelch et al. Fig. S2

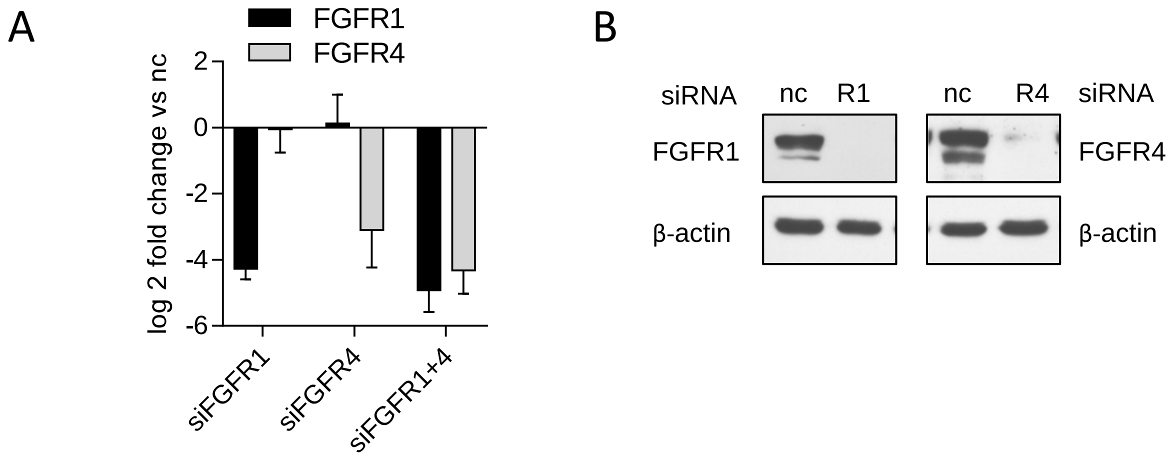

Fig. S3: (A) Target gene expression analysis via qPCR 24 h after transfection with FGFR1, FGFR4 or both siRNAs (10 nM) compared to non coding control (nc) in SPC212, P31 and MSTO cells. **(B)** Western blot analysis of FGFR1 (R1) and FGFR4 (R4) knock down in SPC212 cells 72 h after transfection with respective siRNAs.

Schelch et al. Fig. S3

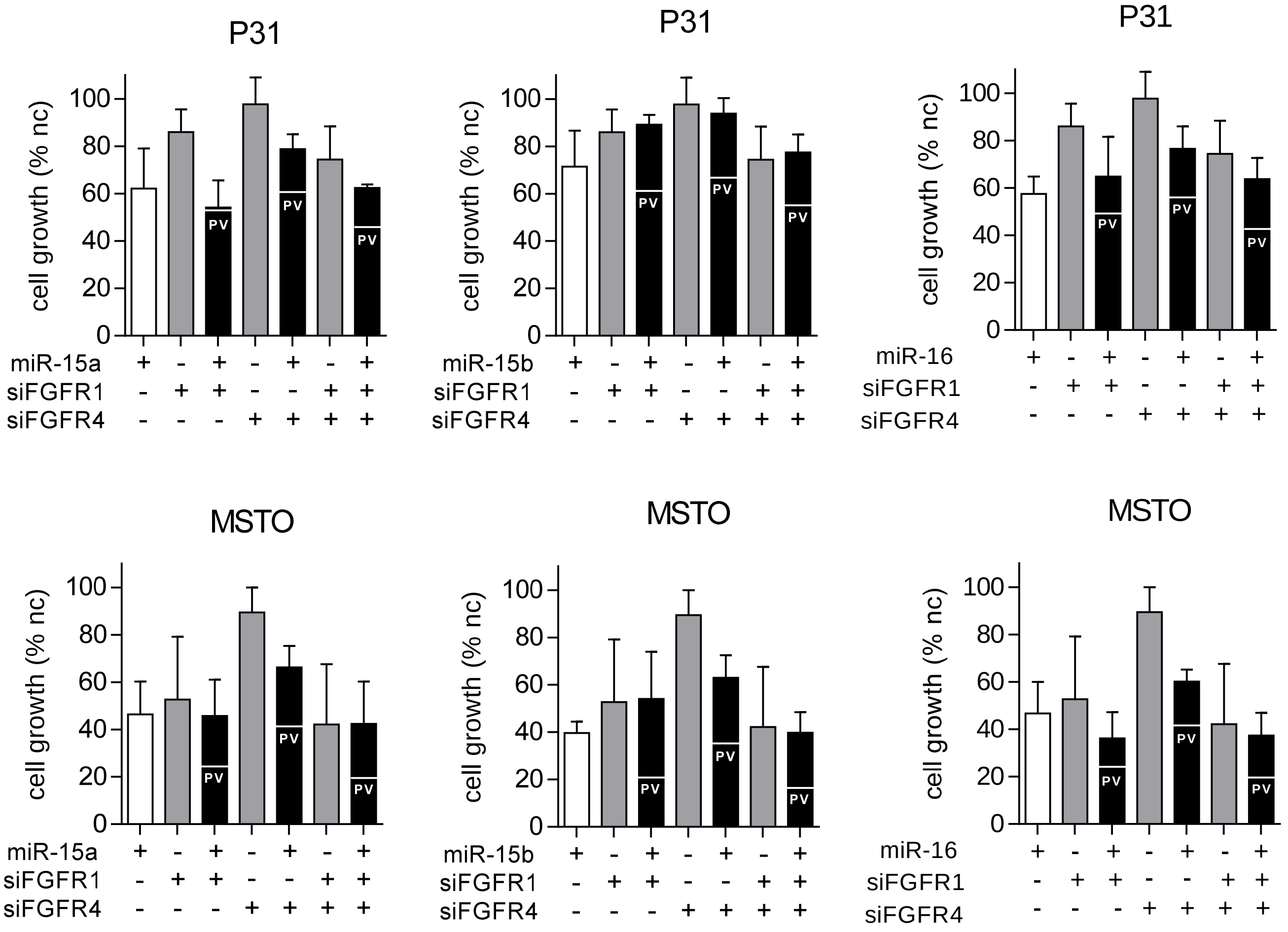

Fig.S4: Growth inhibition assays of P31 and MSTO cells 72 h after transfection with microRNA mimics (5 nM) and/or siRNAs (10 nM) or respective controls (nc). Predicted values of additive interaction (PV) are indicated as white lines within the black combination bars.

Schelch et al. Fig. S4

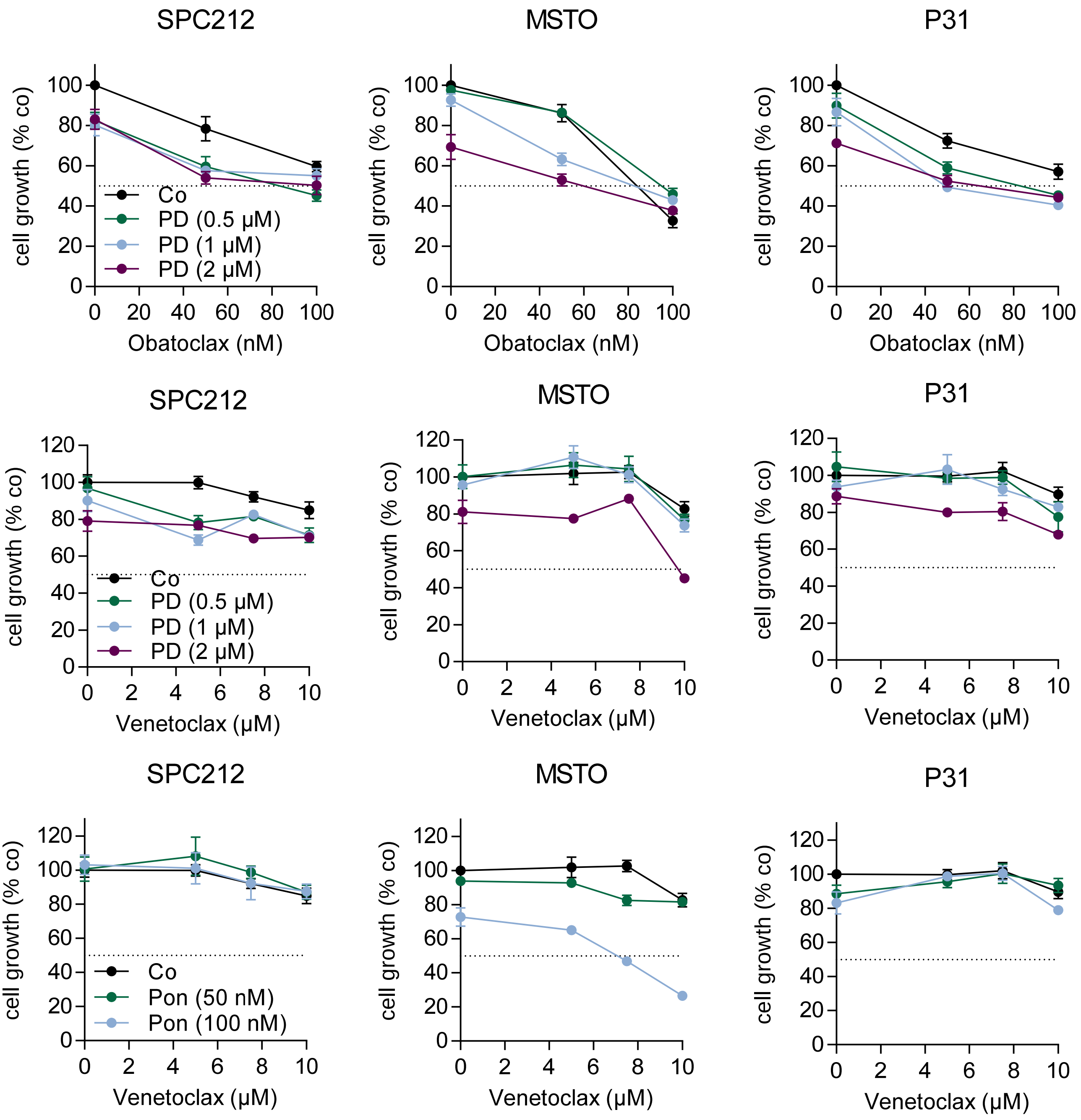

Fig.S4: Growth inhibition assays of SPC212, MSTO and P31 cells 72 hours after treatment with PD166866 (PD) or ponatinib (Pon) in combination with obatoclax or venetoclax, or respective controls (co), at the indicated doses.

Schelch et al. Fig. S5
