# Supplementary material for: A link between the fibroblast growth factor axis and the miR‐16 family reveals potential new treatment combinations in mesothelioma
Source: Mol Oncol. 2017 Nov 18;12(1):58–73. doi: 10.1002/1878-0261.12150 (PMC5748487; doi:10.1002/1878-0261.12150)
Supplement: Supplementary file 2 — Table S1. List of used cell lines, the histological MPM subtype they were derived from, standard growth media and sources. Table S2. List of mimics and siRNA, their sequences/IDs and sources. Table S3. List of cytokines and drugs, their targets and sources. Table S4. Transfection schemes for different assay formats. Table S5. List of primers and probes used in TaqMan‐based reverse transcription and qPCR. Table S6. Primers and their sequences used for SYBR green‐based qPCR. Table S7. Primers and their sequences used for Luciferase reporter assays. Table S8. Fold change, standard deviation and P‐values for microRNAs of individual cell lines after treatment with FGF2. [file MOL2-12-58-s002.docx]

**Supporting information**

Schelch et al., The FGF-Axis and miR-16 in Mesothelioma

**Table S1:** List of used cell lines, the histological MPM subtype they were derived from, standard growth media and sources.

| **Cell line** | **Histotype** | **Growth medium** | **Source** |
| --- | --- | --- | --- |
| LP9 | normal mesothelium | M199 10% FCS + additives* | ATCC |
| Met5a | normal mesothelium | DMEM 10% FCS | ATCC |
| M38K | biphasic | RPMI 10% FCS | University of Helsinki |
| SPC111 | biphasic | RPMI 10% FCS | University of Zürich |
| SPC212 | biphasic | RPMI 10% FCS | University of Zürich |
| P31 | epithelioid | DMEM 10% FCS | Umea University |
| P31 cis | epithelioid | DMEM 10% FCS | Umea University |
| VMC20 | epithelioid | RPMI 10% FCS | Medical University Vienna |
| MM05 | biphasic | RPMI 10% FCS | University of Queensland |
| MM05 cis | biphasic | RPMI 10% FCS | ADRI, Sydney |
| MM05 gem | biphasic | RPMI 10% FCS | ADRI, Sydney |
| MSTO | biphasic | RPMI 10% FCS | ATCC |
| MSTO cis | biphasic | RPMI 10% FCS | ADRI, Sydney |
| MSTO gem | biphasic | RPMI 10% FCS | ADRI, Sydney |

* 3.3 nM EGF, 400 nM hydrocortisone, 870 nM insulin

**Table S2:** List of mimics and siRNA, their sequences/IDs and sources.

| **Mimic/siRNA** | **Sequence/ID** | **Source** |
| --- | --- | --- |
| nc | 5‘ UUGUACUACACAAAAGUACUG 3‘ | GenePharma |
| miR-15a | 5‘ CAAACCAUUAUGUGCUGCUAUU 3‘ | GenePharma |
| miR-15b | 5‘ UAAACCAUGAUGUGCUGCUAUU 3‘ | GenePharma |
| miR-16 | 5‘ CCAAUAUUUACGUGCUGCUAA 3‘ | GenePharma |
| c-81 | 5‘ CCACACGAGUCUUACCAAGUUGCUU 3‘ | GenePharma |
| RRM1 | 5’ AAGAUCUGCUUAUUCAGUAACUGGG 3’ | GenePharma |
| FGFR1/Flg | sc-29316 | Santa Cruz |
| FGFR4 | sc-35368 | Santa Cruz |

**Table S3:** List of cytokines and drugs, their targets and sources.

| **Compound** | **Target** | **Source** |
| --- | --- | --- |
| recombinant human FGF2 | FGFR 1-4 | Sigma |
| PD166866 | FGFR1 | Pfizer |
| Ponatinib | FGFR1, VEGFR2, PDGFRA, Bcr-Abl | Selleck |
| Venetoclax | bcl-2 | Selleck |
| Obatoclax Mesylate | bcl-2, bcl-X_L_, bcl-w, mcl-1 | Selleck |

**Table S4:** Transfection schemes for different assay formats.

| **Format** | **RNA (µl)** | **Lipofectamine (µl)** | **Cells (µl)** | **Final Volume (µl)** | **Cells/well** |
| --- | --- | --- | --- | --- | --- |
| 96-well | 10 | 10 | 100 | 120 | 2.5x10^3^ |
| 6-well | 300 | 300 | 3000 | 3600 | 1.5x10^4^ |
| T25 flask | 800 | 800 | 6400 | 8000 | 4x10^5^ |

**Table S5**: List of primers and probes used in TaqMan-based reverse transcription and qPCR.

| **Target** | **Attribute** | **ID** |
| --- | --- | --- |
| FGFR1 | Probe | Hs 00915142 m1 |
| FGFR4 | Probe | Hs 00242558 m1 |
| FGF1 | Probe | Hs 01092738 m1 |
| FGF2 | Probe | Hs 00960934 m1 |
| FGF5 | Probe | Hs 00738132 m1 |
| FGF18 | Probe | Hs 00826077 m1 |
| 18S | Probe | Hs 99999901 s1 |
| pri-miR-15a | Probe | Hs 03302582 |
| pri-miR-15b | Probe | Hs 03294999 |
| miR-15a | RT-primer (5x) | 000389 |
| miR-15a | Probe (20x) | 000389 |
| miR-15b | RT-primer (5x) | 000390 |
| miR-15b | Probe (20x) | 000390 |
| miR-16 | RT-primer (5x) | 000391 |
| miR-16 | Probe (20x) | 000391 |
| miR-23a | RT-primer (5x) | 000399 |
| miR-23a | Probe (20x) | 000399 |
| miR-24 | RT-primer (5x) | 024 |
| miR-24 | Probe (20x) | 024 |
| miR-93 | RT-primer (5x) | 00109 |
| miR-93 | Probe (20x) | 00109 |
| miR-103 | RT-primer (5x) | 00439 |
| miR-103 | Probe (20x) | 00439 |
| miR-137 | RT-primer (5x) | 001129 |
| miR-137 | Probe (20x) | 001129 |
| miR-191 | RT-primer (5x) | 002299 |
| miR-191 | Probe (20x) | 002299 |
| miR-193a-3p | RT-primer (5x) | 002250 |
| miR-193a-3p | Probe (20x) | 002250 |
| miR-195 | RT-primer (5x) | 000494 |
| miR-195 | Probe (20x) | 000494 |
| miR-223 | RT-primer (5x) | 002295 |
| miR-223 | Probe (20x) | 002295 |
| miR-424 | RT-primer (5x) | 000604 |
| miR-424 | Probe (20x) | 000604 |
| RNU6B | RT-primer (5x) | 001093 |
| RNU6B | Probe (20x) | 001093 |

**Table S6:** Primers and their sequences used for SYBR green-based qPCR.

| **Target** | **Direction** | **Sequence** |
| --- | --- | --- |
| BCL-2 | forward | AATTGCCAAGCACCGCTTC |
|  | reverse | TTCCATCCGTCTGCTCTTC |
| 18S | forward | GGACAAGTGGCGTTCAG |
|  | reverse | GAGCCAGTCAGTGTAGC |

**Table S7:** Primers and their sequences used for Luciferase reporter assays.

| **Target** | **Direction** | **Sequence** |
| --- | --- | --- |
| FGFR1/1 | forward | ATCG CTCGAG AACAAAAAAGAAAAAAAAGGA |
|  | reverse | ATCG GCGGCCGC TAGGAAGAAGAGGTTACAAGG |
| *mutFGFR1/1 | forward | CCCTCAATAAAAATTGC**GAA**TGCTTCATTTATCTA |
|  | reverse | TAGATAAATGAAGCA**TTC**GCAATTTTTATTGAGGG |
| FGFR1/2 | forward | ATCG CTCGAG CTCCTCAGTCGCTATATTAAA |
|  | reverse | ATCG GCGGCCGC AGGTGCTATTTACAGAGAGAA |
| *mutFGFR1/2 | forward | AGGGAAAATGGGAT**GAA**TGCTTTAAATTTCTGAGC |
|  | reverse | GCTCAGAAATTTAAAGCA**TTC**ATCCCATTTTCCCT |
| FGFR4 | forward | ATCG CTCGAG CCTCCTGCCTCCCAAT |
|  | reverse | ATCG GCGGCCGC CCAGGCACACAGAGCTTT |
| FGF1 | forward | ATCG CTCGAG ATTAAGGTGCCTACTGTGTG |
|  | reverse | ATCG GCGGCCGC GACATTTTGATAGGGGTTTA |
| FGF2 | forward | ATCG CTCGAG ACCACTGTAAATTCAAGAAGC |
|  | reverse | ATCG GCGGCCGC GAAAACTGATCAAACACCTC |
| FGF5 | forward | ATCG CTCGAG TTCAAGAAAATAGCTATAATACCT |
|  | reverse | ATCG GCGGCCGC TAGAGATATGCTGGGGTTTAA |
| FGF18 | forward | ATCG CTCGAG TGCAGTCATTTATTTATTGTCC |
|  | reverse | ATCG GCGGCCGC TGCAATATACAGAGAGGTGAAA |

* Mutated nucleotides are shown in bold. The actual microRNA binding site is TGCTGCT which has been replaced with **GAA**TGCT (see forward primer).

**Table S8:** Fold change, standard deviation and P-values for microRNAs of individual cell lines after treatment with FGF2. * P<0.05, ** P<0.01, *** P<0.001

| **Cell line** | **Mean Fold Change** | **SD** | **P-value** |
| --- | --- | --- | --- |
| Target: primiR-15a | | | |
| SPC111 | 0.754908 | 0.494302 | 0.952111 |
| **SPC212** | **0.543789** | **0.133929** | **0.00891 **** |
| **M38K** | **0.192696** | **0.194379** | **0.011617 *** |
| **P31** | **0.401726** | **0.094338** | **0.00341 **** |
| VMC20 | 1.466986 | 0.496646 | 0.25436 |
| MSTO | 0.78737 | 0.386634 | 0.514087 |
| Target: primiR-15b | | | |
| **SPC111** | **0.551499** | **0.133401** | **0.020361 *** |
| **SPC212** | **0.55571** | **0.150586** | **0.014003 *** |
| **M38K** | **0.49401** | **0.59552** | **0.435161 *** |
| **P31** | **0.338902** | **0.238605** | **0.033871 *** |
| **VMC20** | **0.696846** | **0.078539** | **0.013977 *** |
| MSTO | 1.166388 | 0.363246 | 0.552425 |
| Target: miR-15a | | | |
| **SPC111** | **0.637925** | **0.172571** | **0.041258 *** |
| **SPC212** | **0.617997** | **0.163268** | **0.029685 *** |
| **M38K** | **0.36522** | **0.155888** | **0.00451 **** |
| P31 | 0.661195 | 0.256824 | 0.135512 |
| VMC20 | 0.740764 | 0.335148 | 0.335458 |
| MSTO | 0.969849 | 0.32208 | 0.901068 |
| Target: miR-15b | | | |
| SPC111 | 0.658136 | 0.183972 | 0.058311 |
| **SPC212** | **0.500541** | **0.196456** | **0.042122 *** |
| **M38K** | **0.400672** | **0.275041** | **0.036873 *** |
| **P31** | **0.683261** | **0.080027** | **0.005001 *** |
| VMC20 | 0.869447 | 0.192935 | 0.39278 |
| MSTO | 0.987492 | 0.123233 | 0.892801 |
| Target: miR-16 | | | |
| SPC111 | 0.714645 | 0.184349 | 0.09379 |
| SPC212 | 0.782832 | 0.242368 | 0.273845 |
| **M38K** | **0.233707** | **0.112362** | **0.00276 **** |
| **P31** | **0.51071** | **0.144299** | **0.008678 *** |
| VMC20 | 0.860281 | 0.116528 | 0.165192 |
| MSTO | 0.928627 | 0.213234 | 0.660635 |
| Target: miR-23a | | | |
| SPC111 | 0.609442 | 0.207784 | 0.086041 |
| SPC212 | 0.614152 | 0.258011 | 0.138467 |
| M38K | 1.002362 | 0.275655 | 0.991548 |
| **P31** | **0.465459** | **0.160946** | **0.021039 *** |
| VMC20 | 0.860283 | 0.082754 | 0.108408 |
| **MSTO** | **1.976373** | **0.34402** | **0.031837 *** |
| Target: miR-24 | | | |
| **SPC111** | **0.648706** | **0.064764** | **0.001553 **** |
| SPC212 | 0.681763 | 0.194046 | 0.081197 |
| **M38K** | **0.253608** | **0.175836** | **0.010734 *** |
| **P31** | **0.562845** | **0.094184** | **0.002787 **** |
| **VMC20** | **0.693816** | **0.143559** | **0.039308 *** |
| MSTO | 1.08069 | 0.26532 | 0.689286 |
| Target: miR-195 | | | |
| **SPC111** | **0.63778** | **0.152309** | **0.028219 *** |
| SPC212 | 0.629229 | 0.210071 | 0.067049 |
| **M38K** | **0.42442** | **0.241549** | **0.028047 *** |
| **P31** | **0.602573** | **0.129532** | **0.012262 *** |
| VMC20 | 0.922625 | 0.163632 | 0.540284 |
| MSTO | 1.080487 | 0.193347 | 0.587691 |
| Target: miR-223 | | | |
| **SPC111** | **0.39773** | **0.045465** | **0.000388 ***** |
| SPC212 | 0.878288 | 0.27113 | 0.58947 |
| M38K | 0.909101 | 0.141097 | 0.450985 |
| **P31** | **0.453138** | **0.066566** | **0.001599 **** |
| **VMC20** | **0.443028** | **0.035068** | **0.000226 ***** |
| **MSTO** | **0.531121** | **0.032287** | **0.000295 ***** |
| Target: miR-424 | | | |
| **SPC111** | **0.494453** | **0.020613** | **6.17E-05 ***** |
| **SPC212** | **0.410379** | **0.093038** | **0.000858 ***** |
| M38K | 0.904291 | 0.182876 | 0.533156 |
| **P31** | **0.452087** | **0.154907** | **0.007479 *** |
| VMC20 | 0.742332 | 0.17088 | 0.136255 |
| MSTO | 0.844059 | 0.201118 | 0.374683 |
| Target: miR-93 | | | |
| **SPC111** | **0.857908** | **0.035159** | **0.012308 *** |
| **SPC212** | **0.874346** | **0.019177** | **0.003101 **** |
| **M38K** | **0.880504** | **0.036122** | **0.021264 *** |
| P31 | 0.906038 | 0.137643 | 0.427271 |
| **VMC20** | **0.793839** | **0.053105** | **0.013757 *** |
| **MSTO** | **0.840773** | **0.031961** | **0.00683 *** |
| Target: miR-103 | | | |
| SPC111 | 0.988811 | 0.318314 | 0.962735 |
| SPC212 | 1.673239 | 0.814316 | 0.348263 |
| M38K | 1.461821 | 0.726547 | 0.456446 |
| P31 | 0.811139 | 0.143132 | 0.135451 |
| VMC20 | 1.536323 | 0.638865 | 0.300825 |
| MSTO | 1.430827 | 0.846032 | 0.54351 |
| Target: miR-137 | | | |
| SPC111 | 0.953053 | 0.037553 | 0.151802 |
| SPC212 | 0.903462 | 0.32563 | 0.69656 |
| M38K | 0.796959 | 0.349479 | 0.492544 |
| P31 | 0.887314 | 0.134799 | 0.343729 |
| VMC20 | 0.877422 | 0.058697 | 0.067752 |
| MSTO | 1.063662 | 0.040434 | 0.125086 |
| Target: miR-193-3p | | | |
| SPC111 | 0.999118 | 0.40297 | 0.997841 |
| SPC212 | 1.652622 | 0.465157 | 0.118237 |
| M38K | 1.460654 | 0.210351 | 0.060603 |
| **P31** | **0.728493** | **0.110671** | **0.046029 *** |
| VMC20 | 1.161171 | 0.077678 | 0.068778 |
| **MSTO** | **1.172445** | **0.044569** | **0.013885 *** |
